# Knowledge and Education as Barriers and Facilitators to Nicotine Replacement Therapy Use for Smoking Cessation in Pregnancy: A Qualitative Study with Health Care Professionals

**DOI:** 10.3390/ijerph16101814

**Published:** 2019-05-22

**Authors:** Ross Thomson, Lisa McDaid, Joanne Emery, Felix Naughton, Sue Cooper, Jane Dyas, Tim Coleman

**Affiliations:** 1 Division of Primary Care, University of Nottingham, Tower Building, University Park, Nottingham NG7 2RD, UK; sue.cooper@nottingham.ac.uk (S.C.); manerba.it@hotmail.co.uk (J.D.); tim.coleman@nottingham.ac.uk (T.C.); 2 School of Health Sciences, University of East Anglia, Norwich NR4 7UL, UK; L.Mcdaid@uea.ac.uk (L.M.); Joanne.Emery@uea.ac.uk (J.E.); f.naughton@uea.ac.uk (F.N.)

**Keywords:** smoking cessation, pregnancy, nicotine replacement therapy, NRT, stop smoking services

## Abstract

Smoking during pregnancy is a leading cause of negative pregnancy and perinatal outcomes. While UK guidelines recommend nicotine replacement therapy (NRT) for smoking cessation during pregnancy, adherence to NRT is generally low and may partially explain why NRT appears less effective in pregnancy compared to non-pregnant smokers. This study aimed to identify and describe factors associated with NRT adherence from a health professional’s perspective. Two focus groups and one expert group were conducted with 26 professionals involved in antenatal stop smoking services and the data were analysed thematically using a template methodology. From our analyses, we extracted two main themes: (i) ‘Barriers to NRT use in pregnancy’ explores the issues of how misinformation and unrealistic expectations could discourage NRT use, while (ii) ‘Facilitators to NRT use in pregnancy’ describes the different information, and modes of delivery, that stop smoking professionals believe will encourage correct and sustained NRT use. Understanding the barriers and facilitators to improve NRT adherence may aid the development of educational interventions to encourage NRT use and improve outcomes for pregnant women wanting to stop smoking.

## 1. Introduction

Smoking during pregnancy is a leading cause of adverse prenatal outcomes, including miscarriage [1], stillbirth [2], and prematurity [3]. It is also associated with a wide range of childhood health problems [4]. In the United Kingdom (UK), it is estimated that around 11% of women smoke throughout pregnancy, with rates rising considerably with increasing social deprivation [5]. The UK National Institute for Health and Care Excellence (NICE) guidance recommends that through discussion and carbon monoxide (CO) testing (a non-invasive biochemical method for helping to assess whether or not someone smokes), all pregnant women identified as a smoker should be referred to specialist cessation services unless they decline or ‘opt-out’ [6]. Most pregnant smokers are motivated to quit [7] and specialist Stop Smoking Services (SSSs) in England offer these women free cessation support, although up to 75% of women who are referred to SSSs do not take up these appointments [8,9]. Most women taking up the use of this support are offered and initiate the use of nicotine replacement therapy (NRT) [10]. While NRT is a well-established, effective cessation treatment in non-pregnant smokers [11], evidence for NRT efficacy in pregnancy is weaker, with trials demonstrating at best borderline efficacy when pooled [10]. However, there is expert consensus in that nicotine provided via NRT is likely to be less harmful than smoking during pregnancy as it avoids other toxins that are inhaled in tobacco smoke [12] and, consequently, it is offered by all stop smoking services in the UK to pregnant women [13,14]. Generally, pregnant smokers’ adherence to NRT is poor [10] and it is this poor adherence that may help to explain why NRT seems less effective in pregnancy [15]. In trials of non-pregnant smokers, participants report adherence levels of up to 94% [16], while trials with pregnant smokers report that only 7 to 30% of participants completed courses of NRT [10]. The reasons for poor adherence to NRT in pregnancy may be explained by a substantial pregnancy-induced acceleration in the rate of nicotine metabolism [17], which may result in pregnant women needing higher doses of NRT. However, qualitative work with pregnant smokers also suggests that negative perceptions around NRT use, the experience of using NRT, and safety concerns are all potentially adherence issues [18]. Recent work with stop smoking practitioners and experts have highlighted opportunities for improvements in behavioural [19] and clinical support [20] regarding smoking cessation in pregnant women. These studies did include some aspects of NRT provision, for example, how at an organisation level, the delayed prescription of NRT to pregnant women could risk client disengagement [20] or that insufficient knowledge around NRT may act as a barrier to smoking cessation [19]. While the latter study has highlighted general knowledge around NRT as a potential area for improved support, what is unclear is which specific topics practitioners feel are important to address and how this may influence treatment adherence. There is currently a lack of studies that focus purely on stop smoking professionals’ experience of providing and supporting NRT use in pregnancy and how this may impact on women’s adherence. Given the widespread provision of NRT to pregnant women by stop smoking services [13,14], it is important to increase our understanding of factors, from a Health Care Practitioner (HCP) perspective, that may influence pregnant smokers’ adherence to NRT during a quit attempt. To this end, two focus groups and one expert group were conducted with 26 professionals involved in antenatal stop smoking services. This paper will focus on how knowledge and education from smoking cessation practitioners around NRT may influence NRT use in pregnant women who smoke.

## 2. Materials and Methods

We conducted two focus groups with stop smoking practitioners and midwives with specialist smoking cessation training. The aim of the focus groups was to understand how pregnant smokers who wish to stop smoking are supported, in particular with regards to practitioner’s discussions around NRT and how they encouraged NRT adherence. This would give us insight into how issues around NRT provision are dealt with in current practice.

Following the focus groups, we undertook an expert group meeting which was informed by the findings from the focus groups. This expert group included stop smoking service leads and policy makers and explored some of the issues raised in the focus groups in terms of how pregnant women’s experiences of being guided in NRT use can be improved. The findings from these groups will be used to derive content for an intervention to improve pregnant smokers’ adherence to NRT that are acceptable to cessation practitioners and so can be used in UK National Health Service (NHS) behavioural support for stopping smoking.

### 2.1. Participants

Nine participants were recruited for the first focus group (FG1) and 10 participants for the second focus group (FG2) (see Table 1). Participants for FG1 were recruited via email invitations sent out to individuals/organisations in the smoking in pregnancy community or who had previously taken part in other research projects conducted by the group. The potential recruitment of practitioners from other research projects was included as the changing landscape of SSS in the UK made the identification of suitable participant particularly challenging. Participants for FG2 were recruited from a specific stop smoking service known to the research group. Although participants in FG2 had different specialties, all were currently responsible for supporting pregnant women to stop smoking as this was a specific recruitment inclusion criterion for both focus groups. Seven participants were recruited for the expert group (EG) via email invitations (see Table 2). The inclusion criterion for the expert group were that participants should have extensive experience in the field of smoking cessation in pregnancy and/or be in a senior position within a relevant organization. All participants in both focus groups and the expert group came from services in England.

The number of participants in each group is consistent with those recommended in the literature [21,22]. These numbers are considered sufficient to enable a facilitator to allow each person an opportunity to share their views and observations, while not too few as to make it difficult to support a healthy discussion.

Approval for this research was granted by East Midlands—Nottingham 1 NHS Ethics Committee (reference: 12/EM/0388).

### 2.2. Procedure

All participants were provided with a participant information sheet prior to attending the focus or expert group. Those agreeing to participate gave written informed consent on the day of the group.

FG1 was conducted by members of the research team R.T. and L.M. and took place at the University of Nottingham, while FG2 was conducted by L.M. and took place in a private office at the participants’ workplace. Both researchers are experienced in qualitative methods and analysis and have completed formal training on designing and moderating focus groups. The topic guide used in the focus groups (Supplementary Materials S1) was informed by the research team’s previous and ongoing research into smoking and pregnancy with the questions focused on seeking practitioners’ accounts of how they support pregnant smokers who wish to use NRT. The topic guide was applied flexibly across the two focus groups to facilitate a conversational style. Participants were reassured that the researchers had a broad interest in this topic and simply sought out their views while having no preconceived notions regarding best practice. Both focus groups were audio-recorded and transcribed verbatim. FG1 lasted 3 h, while FG2 ran for approximately an hour. This variation was influenced by work schedules as group FG2 was conducted at the end of a stop smoking service team meeting. The focus groups took place in June 2018.

The topic guide used for the expert group (Supplementary Materials S2) was informed by the preliminary findings from the two focus groups alongside the research team’s ongoing interviews with pregnant women who have been offered NRT. While the focus group topic guide was flexible and exploratory in nature, the expert group topic guide was more structured in approach and designed to answer eight specific questions. These questions were devised to elicit what participants believed were the best ways to address the issues raised in previous work and to encourage NRT use. For this reason, it was felt that impartiality on behalf of the facilitator would be important so as not to influence participants’ views towards any suggestions. To mitigate potential bias from the research team, the expert group was facilitated by J.D., who was not part of the research team but has considerable experience in qualitative research. The expert group took place in September 2018, lasted 5 h and was audio-recorded and subsequently transcribed verbatim. Observations and field notes concerning non-verbal behaviours were not collected in either the focus groups or expert group as it was felt that this level of detail was not required for this particular work. This manuscript follows the consolidated criteria for reporting qualitative research (COREQ) checklist for reporting qualitative research [23].

### 2.3. Analysis

The data were analysed thematically [24] using a template methodology [25]. This involved (a) becoming familiar with the issues and subjects raised during the focus groups using the audio recording and/or transcript; (b) initial coding, applying a paraphrase or label (a ‘code’) that describes what has been interpreted in the passage as important; (c) developing an analytical framework (all researchers involved in the analysis agreed on a set of codes to apply to the transcripts based on the initial coding and a-priori theory/themes); (d) applying the analytical framework to the transcripts; (e) interpreting the data and further developing themes and sub-themes. FG1 and FG2 were coded and analysed together. As the expert group was conducted after the focus groups and involved a different topic guide, these data were analysed separately. NVivo 11 software (QSR International Pty Ltd, Melbourne, Australia, released 2015) was used to assist with coding and data management. R.T. initially applied the analytical framework to the transcripts, while L.M. independently reviewed the NVivo output to ensure consistency in coding and analysis, with any disagreement or discrepancy being resolved by discussion and consensus within the research team. We did not undertake any repeat interviews or participant checking due to constraints in the timetable of the overall study, of which these groups were a part, which was due for completion in December 2018. However, findings were discussed with our Patient and Public Involvement (PPI) Advisory Panel, which helped to provide another perspective on the analysis and helped shape the conclusions.

## 3. Results

We extracted two main themes concerning how knowledge and education can act as barriers and facilitators to NRT use in pregnancy. The first theme, ‘barriers to NRT use in pregnancy’, is organised into the sub-themes of ‘listening to misinformation’ and ‘having unrealistic expectations’. The second theme of ‘facilitators to NRT use in pregnancy’ is described in relation to the sub-themes ‘education around NRT’ and ‘methods of support’. In addition to extracts from focus group participants, we also include comments from the expert group to highlight areas where and how improvements might be considered.

Quotes from the focus groups participants will be identified in the following manner: Participant 1 from focus group 1 (P1 FG1), participant 1 from focus group 2 (P1 FG2), participant 1 from the expert group (P1 EG).

### 3.1. Theme 1: Barriers to NRT Use in Pregnancy

In this theme, we explore, from a stop smoking professional’s perspective, how some pregnant women assimilate knowledge about NRT from others and the way this can have a detrimental effect on initiation and sustained NRT use while attempting to stop smoking.

#### 3.1.1. Subtheme 1.1: Listening to Misinformation

Participants felt that some pregnant women had listened to and taken on board misinformation around NRT use in pregnancy that could influence their decision to start and adhere to NRT. When first introduced to using NRT, it was felt women were surprised that they were able to use any medication to help them stop smoking and questioned whether it was safe to use: “Are you sure we’re allowed to use it?” (P5 FG1).

Participants also reported that some women seemed reluctant to try NRT due to preconceived beliefs about the products. The sources of misinformation were varied. Some instances came from the pregnant women’s friends and family:

*P6 FG1  they don’t want NRT because it doesn’t work or so and so used it and this happened or so and so said,* *…‘you know, my mum tried it and it didn’t work’ and that underlying niggling thought constantly gets to them…*

Other instances came from websites, social media or internet forums. It was unclear whether they had used these on-line sources to find out about NRT or whether they were used for general pregnancy support, but the group consensus was that these sources were not particularly helpful in providing accurate information about NRT use in pregnancy.

It was also noted that while coming into contact with different health care practitioners during their pregnancy could provide opportunities to reinforce the stop smoking message, those other health care professionals were sometimes a source of misinformation:

*P2FG2  We still get... that ‘the midwife said that I shouldn’t use a patch, it’s dangerous for me to use a patch because it’ll make the baby stressed’*.

Expert group participants highlighted how previous experiences may inform or misinform women’s NRT use. For example, a previously unsuccessful NRT-assisted quit attempt or one where side effects were insufficiently managed could present a barrier to subsequent use:

*P2 EG  I would explore their past experiences of using NRT because that* *plays a big part and often their contacts who’ve used NRT often influence their decision and that’s an important thing…*

However, there was a sense that women will pick up on the message that suits them at the time. This includes ignoring the advice of trained health care professionals in favour of the lay and potentially dangerous advice proffered by friends and family:

*P4 EG  Sometimes I think they’re going to listen to the person that says what they want to hear… [general murmurs of agreement]… So even if they know they should be listening to their GP rather than their grandma they’re still,* *if the grandma’s turning round saying ‘it’s alright you smoke 20 a day you’ll be fine’, that’s what they want to do. So I think a lot of them will just latch onto that, one person that says what they want to carry on doing really.*

In order to address the impact of misinformation on women’s views and beliefs, practitioners stressed the importance of women receiving consistent, factual messages from all health care professionals that interact with pregnant women. This would also allow different health care practitioners to address issues around misinformation as they arise rather than it being the sole responsibility of stop smoking practitioners.

#### 3.1.2. Subtheme 1.2: Having Unrealistic Expectations

The participants in the focus groups explained that having unrealistic expectations about the role of NRT products in quitting smoking was a particular barrier to women using the product long enough to reduce cravings. They reported that some women believe that just taking the NRT alone will be sufficient to stop them from smoking:

*P8 FG1  I think as well though sometimes with NRT they think it is like the magic pill,* *that they don’t have to put that motivation and effort into it and it’s just going to magically make them not want to smoke and the cravings are going to go…*

Underestimating the role of willpower and how long they would have to use NRT products can result in women not using the products correctly or for long enough. One participant thought that this was a particular problem in women who bought over-the-counter NRT and received no other support.

Not using the products as directed can contribute to women struggling to stay abstinent from smoking and may even result in lapses in cessation or indeed giving up on using NRT altogether:

*P5 FG1  [they have got to] work with it and then if it doesn’t* *work the first instance some people just won’t bother again.*

The expert group recognised the importance of willpower and motivation in a quit attempt and questioned whether women sometimes feel pressured to attempt a quit. In these cases, they questioned whether prescribing NRT was the correct thing to do and that maybe doing so could be counterproductive:

*P5 EG  I think sometimes women do just kind of maybe say yes, whether that be from pressure or whether that be that they are motivated at that moment,* *but then they struggle the following week and they feel embarrassed or ashamed to come back…and that maybe NRT should not be prescribed until women have a more realistic idea of what is involved.*

### 3.2. Theme 2: Facilitators to NRT Use in Pregnancy

This second theme is organised into the sub-themes of ‘education around NRT’ and ‘methods of support’ and describes the different information, and modes of delivery, that stop smoking professionals believe will encourage correct and sustained NRT use.

#### 3.2.1. Subtheme 2.1 Education around NRT

There were different topics that practitioners covered with clients to facilitate NRT use and try to mitigate any problems that may result in women disengaging from their attempt to quit smoking. These include reassurance around the safety of using NRT in pregnancy as they reported that women were unclear whether nicotine could harm the baby:

*P1 FG2  And also it’s really important to try and stress to them that nicotine from what we know like from all the studies, it doesn’t do damage to the baby, whereas all of the chemicals in cigarette smoke and everything else,* *that is the thing that does all the damage.*

Practitioners found that explaining to women how pregnancy increases nicotine metabolism and how they would need to use high doses of NRT was an important message to convey. However, it could be difficult with clients who were particularly worried about NRT safety, noting that “that’s where there’s a bit of resistance” (P5 FG2). This concern over NRT safety may be related to the reasons pregnant women are generally reluctant to take any medications when pregnant. It was felt particularly difficult to explain to women that although NRT is provided on prescription and considered a medical product, it is safe for use during pregnancy.

Another topic was the possible side effects associated with using different NRT products. While practitioners were open about the possibility of side effects, they attempted to reassure women by explaining that the side-effect from NRT are minor especially when compared to the benefits of using the product:

*P1 FG1  It’s just the side effects are just related to how you use it,* *so the patches it’s going to be a bit of skin irritation, with the oral products it’s going to be a little bit of mouth irritation and things like that, but it’s not anything of major concern really when they put it into perspective for them…*

All of the practitioners in the focus groups provided dual NRT therapy, a slow-release product (i.e., a patch) plus a short acting product (e.g., nicotine gum) to relieve sudden cravings, and said they provided directions for use. This included how different products could be used together, for example, using a short acting product around meal times to reduce the urge to smoke that many women have after eating.

There was a sense that the NRT products given out were not always intuitive to use. The practitioners had built up a considerable repertoire of hints, tips and techniques to using the different products in order to increase their efficacy and minimise any potential side effects:

*P1 FG2  …then those sort of little tips you give them, you know,* *and I’ve had patients where they’ve come back and then gone ‘well this is the best thing in the world now and last time it did nothing’.*

The expert group acknowledged the importance of educating pregnant women with regards to NRT safety and the practicalities of using different nicotine products but also highlighted that this was potentially a lot of information to give to some pregnant women:

*P4 EG  So I think you do have to judge with the person sat in front of you and prioritise what you need to tell them on that particular moment because,* *again, you don’t want them walking away with something that they don’t use properly and it puts them off or they don’t know how to use or…it’s a difficult one.*

#### 3.2.2. Subtheme 2.2: Methods of Support

When giving their opinion on possible approaches for a new NRT adherence intervention, participants identified other available support methods used by different services to educate and support women on NRT and facilitate its use.

One service had tried running Facebook groups where pregnant women could access peer support; this initially proved encouraging with “close-knit friendship groups forming” (P6 FG1), but only lasted 12 months as new membership and interest declined.

Some stop smoking services use a computer-based system to manage patient information which also provides text message support. This text message support has practical uses where practitioners can send out routine reminders or confirmation of appointments, and women can text practitioners if they are having a problem or have run out of patches. One practitioner reported using text messaging to provide support to a client that was particularly anxious and found it difficult to talk on the phone. Communicating by text allowed the client to engage with the support on offer.

However, it was made clear by the expert group that these other methods should ideally be used as an adjunct to face-to-face consultations as opposed to replacing them:

*P1 EG  …All the others are absolutely great but just a personal opinion,* *I think the best way is face to face from a trusted person that knows what they’re talking about.*

While some of these services have commissioned their own websites, there was some caution raised over the quality of the information pregnant women may access on other websites:

*P7 EG  I was just going to say websites are good in some circumstances but as long as we’re confident that they’re accessing the websites we would like them to access,* *that we know the correct accurate information and often their idea of a website might be looking on [an internet forum], which is very opinion based instead of fact based.*

## 4. Discussion

This study reports the experiences and views of stop smoking professionals with regards to factors that may influence pregnant smokers’ initiation and adherence to NRT during a quit attempt. Participants from the UK were from a range of professions who either directly supported pregnant women to stop smoking or were involved in the organisation of those services. The analysis revealed the ways in which knowledge and education may influence NRT use in pregnancy. While misinformation and having unrealistic expectations was considered to act as a barrier to pregnant women starting or adhering to NRT during a quit attempt, education given to pregnant women was perceived to encourage NRT use and reduce the likelihood of women disengaging with stop smoking support.

Many of the practitioners reported being particularly frustrated when women came to consultations with beliefs about NRT based on misinformation. Misinformation can be defined as when knowledge and details about factual matters are not supported by clear evidence and expert opinion [26]. Misinformation and misperceptions (the belief that the misinformation claim is true) exist on a range of health issues and can be detrimental to effective treatment options or preventative health behaviours [27]. Misinformation and the inconsistency of health education has been seen to be detrimental in relation to a variety of public health messages including the uptake of childhood vaccinations [28], knowledge around the safe alcohol drinking levels in pregnancy [29], and understanding the risks and safety related to e-cigarettes [30]. The current study has shown that education may be an important factor in why pregnant women are less successful in adhering to NRT, but also how it can be used to assist women to use NRT effectively.

Practitioners in this study noted that for some women, misinformation came from friends and family, often purporting that NRT had not worked for them previously or for someone they knew. Given that it is often reported that pregnant women do not use NRT as advised by health professionals, with many underutilising their NRT to avoid unwanted side effects or avoid perceived harm [15,18], it is unsurprising that some women are unsuccessful in their quit attempts and perpetuate the belief that NRT does not work. In these cases, understanding the circumstances surrounding why these beliefs are held is paramount to being able to address the issues with a tailored and detailed counter-message.

Studies outside of pregnancy have shown that many smokers hold false beliefs about NRT and are generally misinformed about its efficacy [31,32]. It has been identified that smokers who, during a quit attempt with NRT, endorsed the reason ‘I don’t think I need the patch’ were less successful in stopping smoking compared to those who wore the nicotine patch as directed [33]. This, in turn, may be compounded by the conflicting information given to women who smoke by other health care professionals that they may have contact with during their pregnancy. While stop smoking services in the UK actively promote NRT use in pregnancy [13], throughout the health service there is still inconsistent advice, uncertainty over its licensing for use in pregnancy and a lack of guidance over its prescribing among other HCPs [34]. Being informed by others that NRT is not effective and receiving mixed messages from HCPs will likely negatively influence uptake and adherence to NRT.

Another source of misinformation that practitioners had encountered was the internet. The ubiquity of computers, tablets and smartphones have made the internet and social media a popular source for health information [35]. However, studies have demonstrated that health information on websites is highly variable, with some information being low quality, misleading or even incorrect [36]. While the Web and social media can facilitate misinformation, they could also serve as places in which false information is corrected. This can be achieved either by algorithms to produce “related stories” on sites such as Facebook and so can serve to correct the false information provided in the original post [37], or by other users commenting on posts containing misinformation.

As noted in the expert group, it seems possible that some women may ‘only hear what they want to hear’, focusing on the information that confirms their own beliefs while being less receptive to contradictory information. This may be due to efforts to resolve cognitive dissonance. Cognitive dissonance theory argues that this is aimed at resolving the psychological discomfort produced from the discrepancy between an underlying belief that smoking is harmful and continued smoking behaviour [38]. Disengagement beliefs [39] that distort or downplay threatening information are common among pregnant smokers and can be used to justify continued smoking, potentially hindering efforts to quit [40]. Therefore, it is much easier to resolve the psychological discomfort created from the discrepancy between the need to stop smoking (because of a belief that smoking in pregnancy is harmful) and the experience of it being difficult by focusing on misleading messages about NRT (e.g., that it does not work or that it is harmful in pregnancy) and choosing not to use NRT and continuing to smoke. This makes countering misinformation problematic to address as any corrective information is often dismissed or downplayed before it can have the intended effect of updating beliefs. This has been identified as a particular problem in the case of health information online, where people tend to choose congruent information when given the option [41].

In a similar way, having unrealistic expectations can be the result of misleading and inaccurate messages from many commercial sources such as the pharmaceutical industry and media, which undoubtedly contributes to the development of overly optimistic expectations in some patients [42]. While some previous qualitative research has reported that non-pregnant smokers consider willpower, strength, and motivation as central to successful quitting with or without cessation aids [43], other work has demonstrated that some smokers hold unrealistic expectations about what NRT can achieve and underestimate the role of willpower in order to abstain from smoking [44]. After using NRT, smokers also found that it did not live up to expectations in that the effects were too short lived and weak and could only be viewed as a partial substitute for cigarettes [32]. To confront these unrealistic expectations, it may be helpful if pregnant women understand that, while the treatment will help with some of the nicotine cravings and withdrawal, it is unlikely to remove all unpleasant aspects of stopping smoking. Furthermore, emphasising that NRT is an aid to help stop smoking rather than a pleasurable alternative to tobacco may help women to draw on their determination and efforts to stop. Therefore, it seems that practitioners may need to balance informing women about the necessity of willpower and motivation while espousing the role of NRT in stopping smoking in a way that does not raise expectations that may ultimately be unrealised.

Most pregnant women are much more less likely to use medicines during pregnancy compared to outside of pregnancy, which may impact on their decision to take or adhere to a recommended treatment [45]. Pregnant women’s uncertainty about the safety of NRT and the side effects of the different products have been well documented [18,46]. These uncertainties have also been recognised by health care professionals who work with pregnant women [19,47]. In our study, reassuring women about the safety of NRT and the practicalities of using the different products to lessen any side effects was seen to be essential to facilitating its successful use; however, the expert group was mindful that this was a considerable amount of information to give out, and for women to take in, during the initial consultation.

Improved patient education aimed at enhancing patients’ understanding of their treatment, including concerns around safety, along with instructions/techniques around usage is suggested to be adherence enhancing [48]. Practitioners saw that providing pregnant women with this information concerning NRT was important in helping women to initiate and continue its use. While practitioners noted there were different media through which this information could be delivered, there was a general consensus that seeing clients face-to-face was the best way to provide patient education. As noted earlier, the information on NRT use in pregnancy on the Web can be inconsistent at best and smokers seeking quality stop smoking and NRT advice via the internet may have difficulty distinguishing between the numerous web sites available [49].

## 5. Limitations

While the participants in these focus groups were able to provide information about their own practice in their own locality, the provision of stop smoking services to pregnant women is complex and so the findings may not be representative of the UK as a whole. This is especially pertinent as the prevalence of smoking during pregnancy differs both geographically and socioeconomically. In addition, it is worth noting that we have combined the analysis of the focus groups and the expert group, but the aims of the groups were different with the former being exploratory in design, while the latter was more concerned with intervention development.

Although this study reports health professionals’ perceptions of what they consider are the key issues with respect to women starting and continuing to use NRT, this could be different from what women think and what actually influences women to use NRT. In order to address this concern, we have also conducted interviews with pregnant women who have been offered NRT to help them stop smoking, which will also contribute to the design of an intervention to improve NRT adherence.

## 6. Conclusions

The findings have highlighted how the information given to pregnant women who smoke may act as both a barrier and facilitator to them using and adhering to NRT to stop smoking. While education in terms of reassurance about safety and the practicalities of NRT use can aid adherence to NRT, consistent factual messages from health care professionals, the media/Web and friends are needed to help mitigate misinformation and unrealistic expectations that may discourage NRT use. Understanding barriers and facilitators to improve NRT adherence will aid us in developing the educational component of an intervention to encourage NRT use and improve outcomes for pregnant women wanting to stop smoking. This qualitative work will contribute to the design of an intervention to improve NRT adherence which is acceptable to cessation practitioners and could be used in NHS behavioural support for stopping smoking.

## Figures and Tables

**Table 1:** Characteristics of focus group participants.

**Focus Group 1**				
**Participant**	**Age**	**Gender**	**Job Title**	**Experience**
1	32	F	Senior health trainer	5–10 years
2	35	F	Smoking in pregnancy specialist	5–10 years
3	65	F	Smoking in pregnancy specialist	>10 years
4	61	F	Lead midwife smoking cessation	>10 years
5	44	F	Stop smoking specialist	>10 years
6	40	F	Stop smoking advisor	1–2 years
7	48	F	Stop smoking advisor	2–5 years
8	60	F	Health improvement specialist	5–10 years
9	33	F	Stop smoking advisor	5–10 years
**Focus Group 2**				
**Participant**	**Age**	**Gender**	**Job Title**	**Experience**
1	29	F	Stop smoking advisor—young people	5–10 years
2	34	M	Stop smoking advisor	5–10 years
3	59	F	Stop smoking advisor—specialist vapes	>10 years
4	56	F	Stop smoking advisor—pregnancy	5–10 years
5	51	F	Stop smoking advisor—young people	<1 year
6	43	M	Stop smoking service team lead	>10 years
7	50	F	Stop smoking advisor—workplaces	5–10 years
8	52	M	Stop smoking advisor—pregnancy lead	5–10 years
9	59	M	Stop smoking advisor	>10 years
10	28	M	Stop smoking advisor—pregnancy	5–10 years

F: Female; M: Male.

**Table 2:** Characteristics of expert group participants.

Participant	Age	Gender	Job Title	Experience
1	35	F	Smoking in pregnancy specialist	>10 years
2	58	F	Research midwife	5–10 years
3	25	F	Pregnancy lead	2–5 years
4	41	F	Senior tobacco control manager public health England	5–10 years
5	55	F	Stop smoking in pregnancy co-ordinator	>10 years
6	38	F	Stop smoking service lead	>10 years
7	37	F	Stop smoking service manager and tobacco control lead	>10 years

F: Female.

## Supplementary Materials

The following are available online at https://www.mdpi.com/1660-4601/16/10/1814/s1, Materials S1: Focus group topic guide; Materials S2: Expert group topic guide.
