# Supplementary material for: Knowledge and Education as Barriers and Facilitators to Nicotine Replacement Therapy Use for Smoking Cessation in Pregnancy: A Qualitative Study with Health Care Professionals
Source: Int J Environ Res Public Health. 2019 May 22;16(10):1814. doi: 10.3390/ijerph16101814 (PMC6571581; doi:10.3390/ijerph16101814)
Supplement: Supplementary file 1 [file ijerph-16-01814-s001.zip › ijerph-498492-supplementary/Supplementary Materials S1.pdf]

Focus Group topic guide

1. Introducing the Topic

- In your consultations do you describe the harms of smoking in pregnancy? If so, how do you do that?
- What kind of reaction do you get when NRT is mentioned?

2. Discussing NRT with pregnant women

- What do you think are the main issues around actually talking about NRT?
- Are some women reluctant to talk about or try NRT? Can you think of any particular examples?
- Are some women keen to talk about or try NRT? Can you think of any particular examples?

3. After women have decided to use NRT or not

- How do you feel when women are ambivalent or decline NRT?
- Why do you think some women decline NRT? What reasons do they give?
- What guides the choice of NRT type? Is there a difference between what you advise and what the women want to use?
- What do women need to know about taking NRT

4. Support required when women are using NRT

- What if any are the issues occurring when clients have been using NRT for a short while?
- What support do you/can you offer women who have been using NRT?
- What type of support seems to work best?
- What support do you think should be available in an ideal world?

5. Concluding questions

- Is there anything that we have not spoken about today that you think is important regarding NRT?
- Of all the things we've discussed today, what would you say are the most important issues?
