# Supplementary material for: Knowledge and Education as Barriers and Facilitators to Nicotine Replacement Therapy Use for Smoking Cessation in Pregnancy: A Qualitative Study with Health Care Professionals
Source: Int J Environ Res Public Health. 2019 May 22;16(10):1814. doi: 10.3390/ijerph16101814 (PMC6571581; doi:10.3390/ijerph16101814)
Supplement: Supplementary file 1 [file ijerph-16-01814-s001.zip › ijerph-498492-supplementary/Supplementary Materials S2.pdf]

Expert Group Topic Guide

<u>Q1. How do you all maximise pregnant women's take up and subsequent engagement with the use of NRT at the first contact they have with smoking cessation services?</u>

<u>Prompts</u>

- Tips
- What works what doesn't work
- Motivation
- Pressure to say yes (from interviews)
- Frequent concerns they tell you about
- Women's expectations

<u>Q1A. How do you encouraging women to start using NRT without putting them off?</u>

<u>Prompts</u>

- Is it a first contact issue?

<u>Q.2.How do you think the giving out of all relevant information on NRT should be managed?</u>

<u>Prompts</u>

- Overload
- when
- Ideal times -drip feed
  - 1 go
  -Relevant points
- Long term management
- Most effective approach? Why?
- How do you answer any questions/concerns?
- Pick up on deviations from advice

<u>Q2A. What methods of delivery of information on NRT are best at these times?</u>

<u>Prompts</u>

- Face-to-face
- Leaflets
- Texts
- Phone call
- Web site
- apps
- other
- strengths and weaknesses of the different methods?
- Ideas on improving

<u>Q.3. How do you go about countering misinformation about NRT in your regular practice?</u>

Q.4: how do you tackle using NRT during pregnant women's brief lapse or full relapse to smoking to help them becoming abstinent from smoking again or to stop smoking permanently?

Prompts

- Advice you give
- Best for giving up completely
- Differences between lapse and relapse?

Q.5 Drawing on your experiences what would you include in a Rolls Royce support package of care for pregnant women on their journey with NRT in-between or after consultations with practitioners?

Prompts

- Key messages to include
- timing
- format
- Practical vs emotional
- content
- Do/should women have choices on delivery
- why

Q.6 Please discuss/share with each other, how you are dealing with this issue of vaping and NRT combined with your clients

Q.7 Are any of you seeing/working on, opportunities in these changes, for improving women's experiences of using NRT?

Prompts

- Technology
- If not, why not
